# Keeping Pace with the Red Queen: Identifying the Genetic Basis of Susceptibility to Infectious Disease

**DOI:** 10.1534/genetics.117.300481

**Published:** 2017-12-08

**Authors:** Ailene MacPherson, Sarah P. Otto, Scott L. Nuismer

**Affiliations:** * Department of Zoology, University of British Columbia, Vancouver, V6T 1Z4, Canada; † Biodiversity Research Centre, University of British Columbia, Vancouver, V6T 1Z4, Canada; ‡ Department of Biological Sciences, University of Idaho, Moscow, Idaho 83844

**Keywords:** host–parasite coevolution, GWAS, *Daphnia magna*, *Pastueria ramosa*, genetic architecture of resistance

## Abstract

Genome-wide association studies are widely used to identify “disease genes” conferring resistance/susceptibility to infectious diseases. Using a combination of mathematical models and simulations, we demonstrate that genetic interactions between hosts and parasites [genotype-by-genotype (G × G) interactions] can drastically affect the results of these association scans and hamper our ability to detect genetic variation in susceptibility. When hosts and parasites coevolve, these G × G interactions often make genome-wide association studies unrepeatable over time or across host populations. Reanalyzing previously published data on *Daphnia magna* susceptibility to infection by *Pasteuria ramosa*, we identify genomic regions consistent with G × G interactions. We conclude by outlining possible avenues for designing more powerful and more repeatable association studies.

## Abstract (precis)

The results of genome-wide association studies are known to be affected by epistasis and gene-by-environment interactions. Using a statistical model....

INFECTIOUS diseases are pervasive. So pervasive, in fact, that without effective mechanisms of resistance, host populations can be quickly reduced in size or even driven to extinction. For instance, chestnut blight effectively wiped out the American chestnut, which had little if any resistance to this novel pathogen, after its introduction to North America in the early 1900s (Anagnostakis 2000; Anderson *et al.* 2004). Similarly, when Myxoma virus was introduced to Australia in the 1950s, local rabbit populations were almost entirely susceptible, resulting in millions of deaths and the decimation of local populations (Ratcliffe *et al.* 1952). Human populations, too, have been heavily affected by infectious disease in the past, perhaps most notably during the 1918 influenza pandemic that killed >50 million people before fading away in 1920 (Johnson and Mueller 2002; Taubenberger and Morens 2006). Although these examples are striking and demonstrate the impact of unchecked infectious disease, they are far from the norm. More commonly, host populations have effective mechanisms of resistance against pathogens they encounter regularly (Revers *et al.* 2014), with significant variability between populations depending on their history of exposure (Bartholomew 1998; Weatherall and Clegg 2002).

The existence of substantial variation in resistance to infectious disease within host populations has generated hope that it may be possible to identify the genes conferring resistance. Identifying such resistance genes would pave the way for genetic engineering of resistant crops and livestock, focus drug development efforts on likely targets, and open the door to gene therapeutic approaches within human populations. As the genomic revolution has progressed, it has become increasingly common to search for these “resistance genes” using genome-wide association studies (GWAS) (Newport and Finan 2011; Rowell *et al.* 2012). Loosely speaking, these studies compare the marker genotypes of individuals infected with disease and those uninfected and ask which loci predict an individual’s infection status. The GWAS approach has now been used to successfully identify a range of candidate genes thought to be important in resistance to infectious disease in plants and animals (Chapman and Hill 2012; Khor and Hibberd 2012; Wang *et al.* 2012; Zila *et al.* 2013; Gurung *et al.* 2014).

Despite the successes of the GWAS approach in some cases, it is becoming increasingly recognized that the approach has significant limitations. For instance, GWAS are most powerful when resistance depends on common genetic variants with relatively large phenotypic effects (Manolio *et al.* 2009). In addition, which candidate genes are identified by this method may depend on the environment in which the study is conducted (Thomas 2010). These limitations apply to GWAS in general, not just those studies focused on infectious disease, and are widely recognized. When GWAS are used to understand the genetic basis of resistance to infectious disease, however, a potentially more important problem arises. Specifically, the resistance genes identified within the host population may depend on the genetic composition of the infectious disease itself (Newport and Finan 2011). This sensitivity of the GWAS approach to the genetic composition of the infectious disease becomes acute any time genotype-by-genotype (G × G) interactions exist; in other words, when particular combinations of host and pathogen genes yield resistance whereas other combinations lead to susceptibility. These G × G interactions may have drastic effects on the results of genetic association studies and our understanding of disease resistance (Lambrechts 2010), similar to the effects of gene-by-environment interactions. One particularly disconcerting possibility is that rapid pathogen evolution or host–pathogen coevolution will cause the host resistance genes that can be identified by GWAS to fluctuate rapidly over time.

Here we quantitatively explore the performance of GWAS when resistance to infectious disease involves G × G interactions between host and disease. We begin by presenting a general mathematical model of an association study to investigate disease resistance and evaluate the role of G × G interactions for several forms of host–parasite interactions. We then simulate host–pathogen coevolution to illustrate the extent to which G × G interactions may vary across time and/or space. We conclude by reanalyzing published genome-wide association data (Bourgeois *et al.* 2017) of *Daphnia magna* resistance to its *Pasteuria ramosa* pathogen, distinguishing regions of the genome associated with overall health from those involved in resistance specific to a particular *P. ramosa* strain.

## Model

We consider a scenario, common in practice, where host resistance is measured as a continuous quantitative trait. This would be the case, for instance, if host resistance is assessed by measuring viral load, duration of infection, or damage to host tissues. Our model assumes that host resistance depends on the value of a quantitative trait in the host, $z_{H},$ relative to the value of a quantitative trait in the pathogen, $z_{P}.$ Specifically, we assume host susceptibility, *S*, is given by the following function:

$$S=f\left(z_{H}-z_{P}\right).$$ (1)

**Figure 1:**
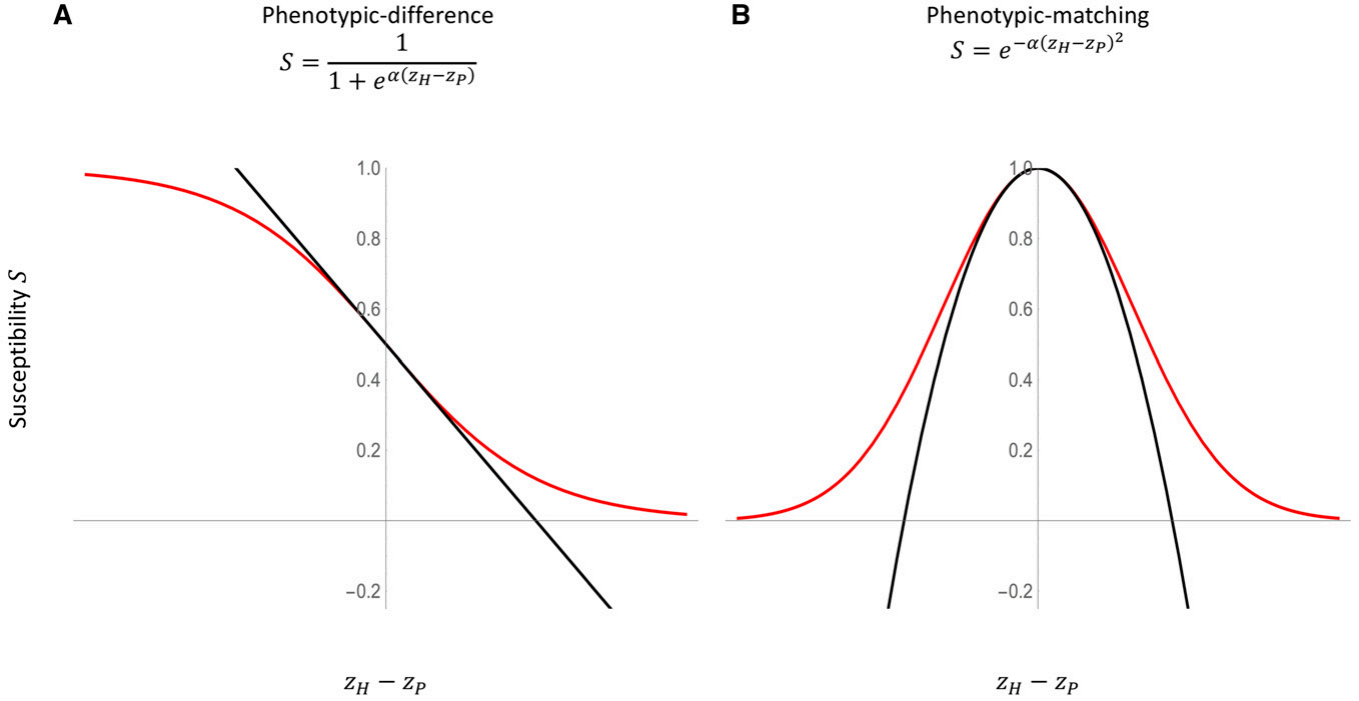
Host–parasite interaction models. Susceptibility to infection as a function of the distance between host and pathogen phenotypes, $z_{H}-z_{P},$ for the (A) phenotypic-difference and (B) phenotypic-matching model. Red curves show exact functions whereas black curves are the quadratic approximations.

The function *f* is sufficiently general to accommodate many commonly observed resistance mechanisms. For instance, in the interaction between the snail *Biomphalaria glabrata* and its trematode parasites, resistance depends on the relative quantities of reactive oxygen molecules in the snail ($z_{H}$) and reactive oxygen scavenging molecules produced by the parasite ($z_{P}$) (Bayne 2009; Mon *et al.* 2011). In cases like these, the function *f* may take a sigmoid form which we call the phenotypic-difference model (Figure 1A) (Nuismer *et al.* 2007; Ashby and Boots 2017):

$$f\left(z_{H}-z_{P}\right)=\frac{1}{1+e^{\alpha \left(z_{H}-z_{P}\right)}}.$$ (2)

In contrast, in the interaction between the schistosome parasite, *Schistosoma mansoni*, and its snail host, *B. glabrata*, resistance depends on the degree to which the conformation of defensive FREP molecules produced by the snail ($z_{H}$) match the conformation of parasite mucin molecules ($z_{P}$) and successfully bind to them (Mitta *et al.* 2012). In such cases, the function *f* may take a Gaussian form which we call a phenotypic-matching model (Figure 1B) (Kopp and Gavrilets 2006):

$$f\left(z_{H}-z_{P}\right)=e^{-\alpha {\left(z_{H}-z_{P}\right)}^{2}}.$$ (3)

To study the effects of genetic interactions on susceptibility to infection, *S*, we must integrate genetics into our phenotypic model. For a haploid host and pathogen where $z_{H}$ and $z_{P}$ depend on $n_{H}$ and $n_{P}$ biallelic loci, respectively, we can write general expressions for these phenotypes as functions of alleles present in each species:

$$\begin{array}{c} z_{H}=b_{H0}+\sum_{i=1}^{n_{H}}b_{Hi}X_{Hi}+\sum_{\begin{array}{c} i,j\\ i\neq j \end{array}}^{n_{H}}b_{Hi,Hj}X_{Hi}X_{Hj}+\sum_{\begin{array}{c} i,j,k\\ i\neq j\neq k \end{array}}^{n_{H}}b_{Hi,Hj,Hk}X_{Hi}X_{Hj}X_{Hk}+\ldots +\epsilon_{H}\\ z_{P}=b_{P0}+\sum_{i=1}^{n_{P}}b_{Pi}X_{Pi}+\sum_{\begin{array}{c} i,j\\ i\neq j \end{array}}^{n_{P}}b_{Pi,Pj}X_{Pi}X_{Pj}+\sum_{\begin{array}{c} i,j,k\\ i\neq j\neq k \end{array}}^{n_{P}}b_{Pi,Pj,Pk}X_{Pi}X_{Pj}X_{Pk}+\ldots +\epsilon_{P} \end{array}$$ (4)

In these expressions, $X_{Mi}$ is an indicator variable describing the allelic state (0 or 1) of an individual of species *M* at locus *i*, $b_{M0}$ is the phenotype of an individual of species *M* with all “0” alleles, and $b_{Mi}$ is the additive effect of carrying a “1” allele at locus *i* in species *M*. The remaining coefficients ($b_{Mi,Mj},$ $b_{Mi,Mj,Mk},$ *etc*.) describe epistatic interactions among loci. Finally, $\epsilon_{M}$ captures an environmental contribution to the phenotype of species *M*, which is assumed to have mean 0, a constant variance, and be uncorrelated with an individual’s phenotype. Substituting Equation 4 into Equation 1 yields a model of host susceptibility as a function of host and pathogen genotypes.

Our goal now is to use this genetic model to predict the sensitivity of GWAS to the genetic composition of the pathogen population. We will explore both traditional, single-species GWAS approaches and a novel approach that takes genetic information from both host and pathogen into account (co-GWAS). Our investigation will rely on a pair of complementary approaches. First, we will develop and analyze analytical approximations that quantify the sensitivity of GWAS and co-GWAS approaches to changes in pathogen genotype frequencies. These analytical approximations will rely on simplified genotype–phenotype maps and will not explicitly integrate evolution and coevolution. Second, we will develop and analyze simulations that allow us to explore the consequences of rapid pathogen evolution and coevolution between the species on the performance of both GWAS and co-GWAS approaches.

## Analytical Approximation

To simplify the genetic model of resistance developed in the previous section sufficiently for mathematical analysis, we begin by considering the case where $n_{H}=n_{P}=2.$ In addition, we assume that the phenotypes of host and pathogen are not too far from one another, such that the quantity $z_{H}-z_{P}$ is small relative to the extent of phenotypic specificity (*α* in Equations 2 and 3). Under this assumption, Equation 1 can be approximated by its second order Taylor series expansion. This allows the genetic model of susceptibility to be simplified to the following approximate expression:

$$\begin{array}{c} S\approx f\left(0\right)+f^{\prime }\left(0\right)\left[\left(b_{H0}+b_{H1}X_{H1}+b_{H2}X_{H2}+\epsilon_{H}\right)-\left(b_{P0}+b_{P1}X_{P1}+b_{P2}X_{P2}+\epsilon_{P}\right)\right]\\ +\frac{1}{2}f^{\prime \prime }\left(0\right){\left[\left(b_{H0}+b_{H1}X_{H1}+b_{H2}X_{H2}+\epsilon_{H}\right)-\left(b_{P0}+b_{P1}X_{P1}+b_{P2}X_{P2}+\epsilon_{P}\right)\right]}^{2}+O\left[{\left(z_{H}-z_{P}\right)}^{3}\right], \end{array}$$ (5)

where primes indicate derivatives with respect to the distance between host and pathogen phenotypes. With (5) in hand, we have a model that predicts host resistance as a function of host and pathogen genotypes. In the following two sections, we will use (5) to investigate how the genetic composition of the pathogen population influences the results of GWAS and co-GWAS. Extending these models to complete G × G association studies requires a large number of pathogen loci ($n_{P}\gg 2$) and thus may be computationally prohibitive. For many pathogens, however, strain type or subtype may be known and capture much of the relevant genetic variation in the pathogen population. In these cases, tracking pathogen types can greatly reduce the effective number of loci, even to $n_{P}=2$ as in Equation 5. Such simplifications should allow us to expand beyond two host loci to a whole host genome ($n_{H}\gg 2$), while avoiding the computational complexity of tracking all possible genetic interactions between the full host genome and the full parasite genome.

### Single-species GWAS

We envision a standard GWAS where susceptibility to infection has been measured for some number of host individuals, each of which has also been genotyped at a large number of marker loci. To focus our model on the effects of species interactions, we will assume this data accurately provides us with the genotype of individuals at the two host resistance loci. Using this data, the goal of the genetic association study is to partition host susceptibility between these genes relative to their effects. This can be done by fitting susceptibility with a linear combination of the genetic indicator variables:

$$S\approx \beta_{H0}+\beta_{H1}X_{H1}+\beta_{H2}X_{H2}+\beta_{H1,H2}X_{H1}X_{H2},$$ (6)

where the *β* coefficients can be found using least squares regression. The biological interpretation of this linear model is straightforward. The intercept coefficient, $\beta_{H0},$ is the expected host resistance when both 0 host alleles are present. The coefficients $\beta_{H1}$ and $\beta_{H2}$ are the inferred additive effects of the 1 alleles at the first and second loci, respectively, and $\beta_{H1,H2}$ captures the epistatic interaction between the two host 1 alleles. Solving for the coefficients in (6) we have (see Supplemental Material, *Mathematica* notebook in File S1):

$$\begin{array}{c} \beta_{H0}=f\left(0\right)+f^{\prime }\left(0\right)\left[{\tilde{b}}_{H0}-\left({\tilde{b}}_{P0}+b_{P1}q_{P1}+b_{P2}q_{P2}\right)\right]+\frac{1}{2}f^{\prime \prime }\left(0\right)\{{\left({\tilde{b}}_{H0}-{\tilde{b}}_{P0}\right)}^{2}+\\ b_{P1}^{2}q_{P1}+b_{P2}^{2}q_{P2}+2\left[\left(b_{P1}q_{P1}+b_{P2}q_{P2}\right)\left({\tilde{b}}_{H0}-{\tilde{b}}_{P0}\right)-b_{P1}b_{P2}\left(q_{P1}q_{P2}+D_{P}\right)\right]\}\\ \beta_{Hi}=f^{\prime }\left(0\right)b_{Hi}+\frac{1}{2}f^{\prime \prime }\left(0\right)\left[b_{Hi}^{2}+2b_{Hi}\left(b_{H0}-b_{P0}-q_{P1}b_{P1}-q_{P2}b_{P2}\right)\right]\\ \beta_{H1,H2}=f^{\prime \prime }\left(0\right)b_{H1}b_{H2}, \end{array}$$ (7)

for *i* = {1,2}, where f(0), $f^{\prime }\left(0\right),$ and $f^{\prime \prime }\left(0\right)$ are the resistance function and its first and second derivative evaluated at 0 as in Equation 5, and where ${\tilde{b}}_{H0}=b_{H0}+\epsilon_{H}$ and ${\tilde{b}}_{P0}=b_{P0}+\epsilon_{P}.$ Importantly, these expressions for the coefficients depend on the allele frequency at the pathogen loci, $q_{P1}$ and $q_{P2},$ as well as the linkage disequilibrium between them, $D_{P}.$ Note that the relevant allele frequencies and linkage disequilibrium are among pathogens to which the host is exposed, which may not be equivalent to the pathogen population as a whole.

**Figure 2:**
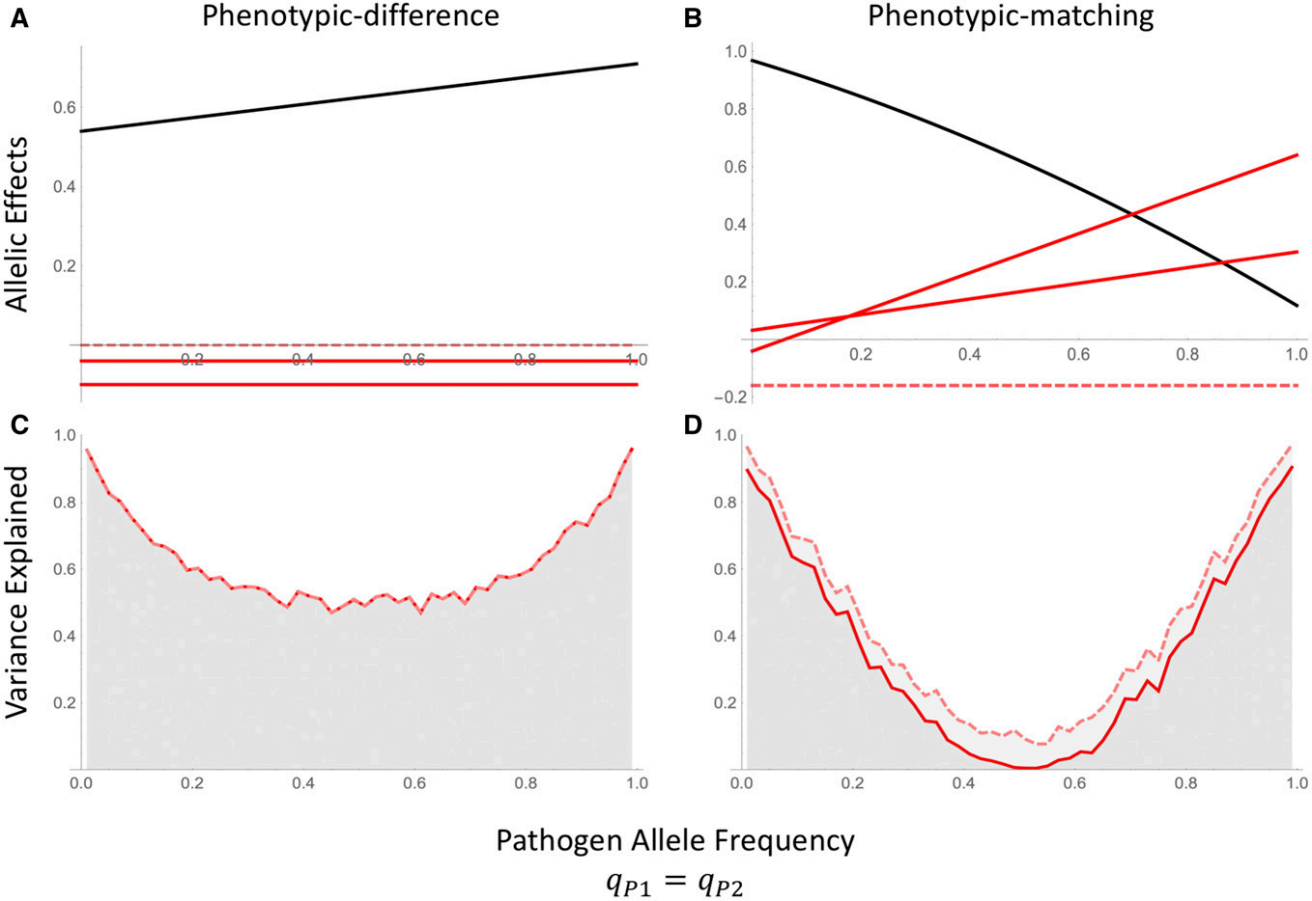
Host-only model with resistance dependent on phenotypic differences (A,C) or phenotypic matching (B,D) between hosts and parasites. (A and B) Allelic effects inferred using the host-only design from Equation 6: $\beta_{0}$ (black), $\beta_{H1}$ and $\beta_{H2}$ (solid red lines), $\beta_{H1,H2}$ (dashed red). (C and D) Variation explained by host additive effects only (solid line), and host additive and epistatic effects (dashed line) as given by the host-only model in (6).

As a result of the dependence of the coefficients in (7) on the pathogen allele frequencies and linkage disequilibrium, the allelic effects (*β*’s) inferred by a host-only GWAS can be quite sensitive to the genetic composition of the pathogen population (Figure 2). Changes in pathogen allele frequency can alter the magnitude and sign of the inferred effects. From a practical standpoint, if susceptibility is assayed in two host populations that are exposed to pathogen populations that differ greatly in their allele frequencies, one may find a host allele has a protective effect in one population but increases risk in the other. Similar to hidden host population structure, uncontrolled differences in the pathogen population can greatly alter the inferences of single-species GWAS.

A second result that can be drawn from Equation 7 is that when the resistance function is approximately linear, $f^{\prime \prime }\left(0\right)=0,$ the inferred additive and epistatic effects, $\beta_{H1},\;\beta_{H2},$ and $\beta_{H1,H2}$ are independent of the pathogen allele frequencies. For example, in contrast to the nonlinear phenotypic-matching model where the inferred effects vary with pathogen allele frequency, the inferred effects remain constant in the approximately linear phenotypic-difference model (Figure 2). A third conclusion from Equation 7 is that, at least under the assumption that $z_{H}-z_{P}$ is small, the epistatic interaction between the host loci, $\beta_{H1,H2},$ is independent of pathogen genetics. We will explore the consequences of this dependence on the pathogen allele frequencies for the stability of GWAS-inferred effects across evolutionary time (See the *Host–Parasite Coevolution* section below).

In addition to identifying the allelic effects on host resistance, an important metric of GWAS performance is the proportion of phenotypic variation explained by the identified causative loci. Given the dependence of the estimated allelic effects on pathogen allele frequencies, we calculated the total phenotypic variation explained by the host loci across the range of pathogen allele frequencies (Figure 2, C and D). When the pathogen population is monomorphic ($q_{P1}=q_{P2}=0\;\text{or}\;1$), the host loci can explain 100% of the genetic variation in the phenotype. If the pathogen population is polymorphic, however, the host-only approach may explain as little as 10% of the variation. Partitioning the total variation explained into the additive and epistatic contributions demonstrates that, due to changes in the additive effect size $b_{Hi},$ the relative contribution of additive and epistatic effects also varies with pathogen allele frequency and depends on the form of the host–parasite interaction.

### Two-species co-GWAS

The results derived in the previous section demonstrate that traditional single-species GWAS may be sensitive to the genetic composition of the pathogen population at loci involved in host–pathogen specificity. In this section, we attempt to overcome this problem by developing an alternative GWAS design in which both host and pathogen genetics are incorporated. In contrast to the traditional method where only host genotypes are recorded, this design requires that both host and pathogen genotypes are known. As with Equation 6, we now attempt to fit host resistance as a linear function of the allelic indicator variables, but we include pathogen indicators as well as interaction terms between host and pathogen loci:

$$\begin{array}{c} S\approx \beta_{0}+\beta_{H1}X_{H1}+\beta_{H2}X_{H2}+\beta_{H1,H2}X_{H1}X_{H2}+\beta_{P1}X_{P1}+\beta_{P2}X_{P2}+\beta_{P1,P2}X_{P1}X_{P2}\\ +\beta_{H1,P1}X_{H1}X_{P1}+\beta_{H1,P2}X_{H1}X_{P2}+\beta_{H2,P1}X_{H2}X_{P1}+\beta_{H2,P2}X_{H2}X_{P2}. \end{array}$$ (8)

As with Equation 6, the coefficients of this equation have straightforward biological interpretations. The intercept, $\beta_{0},$ describes the expected host resistance when all host and pathogen loci have 0 alleles. Terms 2, 3, 5, and 6 describe the additive effects of each individual host and pathogen 1 allele; and terms 4 and 7 describe the epistatic interactions between loci within the host and pathogen, respectively. The remaining four terms describe the G × G interactions between pairs of host and pathogen loci.

Despite the complexity of Equation 8, and hence the logistical and computational challenges of applying it, the expressions for each of these coefficients in terms of the host and pathogen phenotypic effects are simple (see *Mathematica* notebook in File S1):

$$\begin{array}{c} \beta_{H0}=f\left(0\right)+f^{\prime }\left(0\right)\left({\tilde{b}}_{H0}-{\tilde{b}}_{P0}\right)+\frac{1}{2}f^{\prime \prime }\left(0\right){\left({\tilde{b}}_{H0}-{\tilde{b}}_{P0}\right)}^{2}\\ \beta_{Hi}=f^{\prime }\left(0\right)b_{Hi}+\frac{1}{2}f^{\prime \prime }\left(0\right)b_{Hi}\left[b_{Hi}+2\left({\tilde{b}}_{H0}-{\tilde{b}}_{P0}\right)\right]\;\text{for}\;i=\left\{1,2\right\}\\ \beta_{H1,H2}=f^{\prime \prime }\left(0\right)b_{H1}b_{H2}\\ \beta_{Pi}=-f^{\prime }\left(0\right)b_{Pi}-\frac{1}{2}f^{\prime \prime }\left(0\right)b_{Pi}\left[b_{Pi}+2\left({\tilde{b}}_{H0}-{\tilde{b}}_{P0}\right)\right]\;\text{for}\;i=\left\{1,2\right\}\\ \beta_{P1,P2}=f^{\prime \prime }\left(0\right)b_{P1}b_{P2}\\ \beta_{Hi,Pj}=-f^{\prime \prime }\left(0\right)b_{Hi}b_{Pj}\;\text{for}\;i=\left\{1,2\right\},\;j=\left\{1,2\right\}. \end{array}$$ (9)

**Figure 3:**
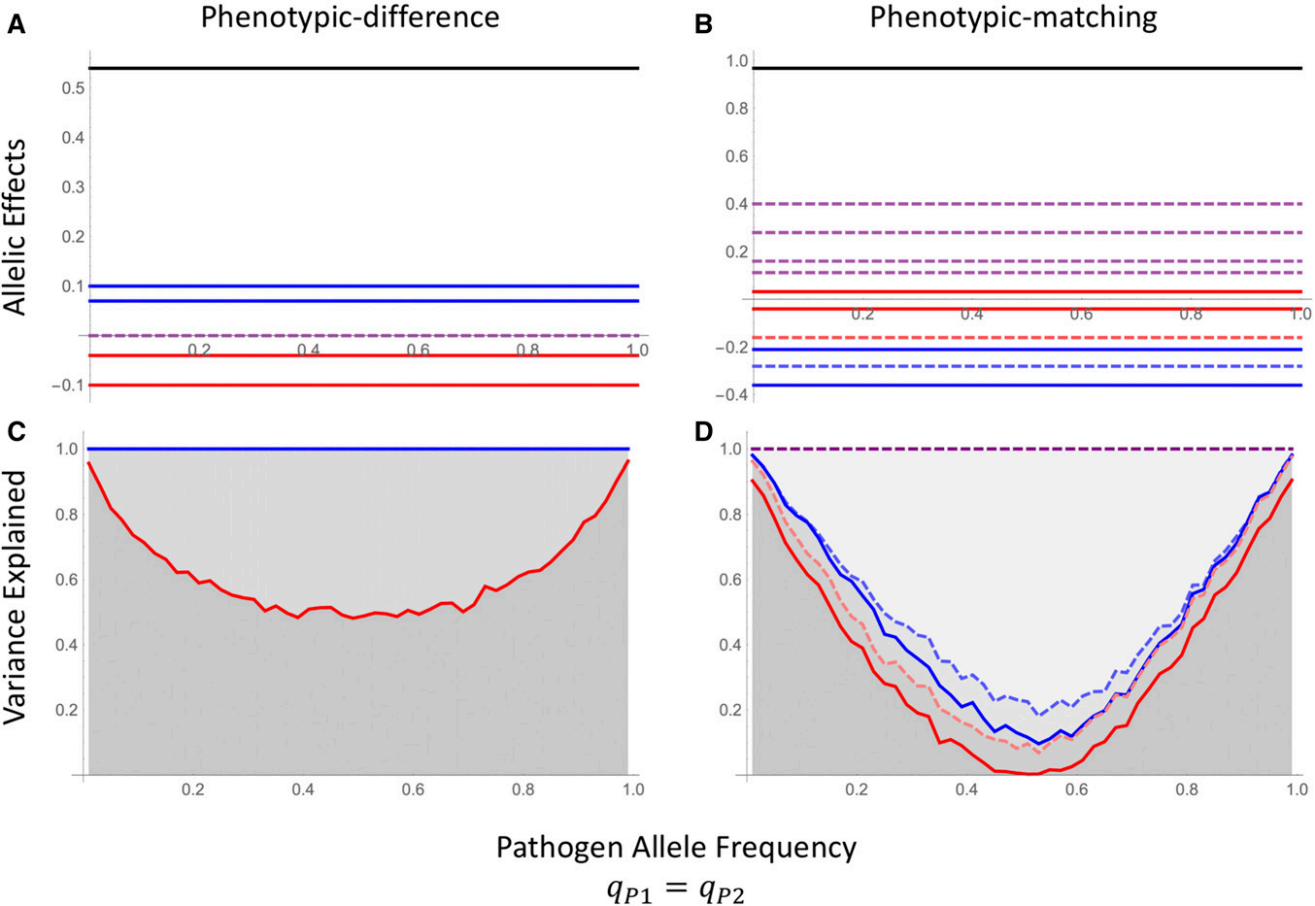
Host–pathogen model with phenotypic-difference (A,C) or phenotypic-matching (B,D) based resistance. (A and B) Allelic effects inferred using the host-parasite design from Equation 8: $\beta_{0}$ (black), $\beta_{Hi}$ (solid red), $\beta_{H1,H2}$ (dashed red), $\beta_{Pi}$ (solid blue), $\beta_{P1,P2}$ (dashed blue), and $\beta_{Hi,Pj}$ (dashed purple). (C and D) Variation explained by host additive effects only (solid red), host additive and epistatic effects (dashed red), host and pathogen additive and epistatic effects (dashed blue), and a full host–pathogen model as given in Equation 8.

Comparing the equations in (9) with the coefficients in (7) reveals an important conclusion: the effect sizes no longer depend on the pathogen allele frequencies nor the linkage disequilibrium (Figure 3, A and B). This result suggests that the two-species, co-GWAS approach is more robust to changes in the genetic composition of the pathogen population and thus may be much less sensitive to rapid evolution and spatial genetic structuring within the pathogen population.

In addition to stabilizing the estimated allelic effects across pathogen allele frequencies, the total phenotypic variation explained by the co-GWAS greatly exceeds that of the host-only GWAS. For the two-locus case explored here, the co-GWAS approach can explain 100% of the variation regardless of pathogen allele frequency (Figure 3, C and D). The contributions of additive, epistatic, and G × G interactions do, however, vary with pathogen allele frequency. As with the host-only approach, when the pathogen population is monomorphic the host effects explain all of the observed phenotypic variation. In summary, unlike the host-only model, the effect size coefficients (Equation 9) and the total variation explained, no longer vary with pathogen allele frequency. This contrast between the host-only and co-GWAS approaches is particularly relevant any time the composition of the pathogen population is likely to differ between the sample used for the association study and the population in which the resulting inferences are applied. In the following section we explore how temporal changes in the host and pathogen populations driven by coevolution affects the reproducibility of GWAS over time and, by extension, space.

## Host–Parasite Coevolution

To simulate host–parasite coevolution, we envision a system where each host comes into contact with a single parasite each generation. The probability that this contact results in infection is determined by host susceptibility, *S*, which is a function of the host and parasite genotype. Infected hosts experience a fitness cost $\xi_{H},$ whereas their infecting parasites receive a fitness benefit $\xi_{P}.$ In the absence of infection, both hosts and parasites have a fitness of 1. Together, these assumptions lead to the following fitness of a host with genotype $\left\{X_{H1},X_{H2}\right\}$ that comes into contact with a pathogen with genotype $\left\{X_{P1},X_{P2}\right\}:$

$$W_{H}=1-\xi_{H}S\left(X_{H1},X_{H2},X_{P1},X_{P2}\right);$$ (10)

whereas the pathogen has a fitness of

$$W_{P}=1+\xi_{P}S\left(X_{H1},X_{H2},X_{P1},X_{P2}\right).$$ (11)

**Figure 4:**
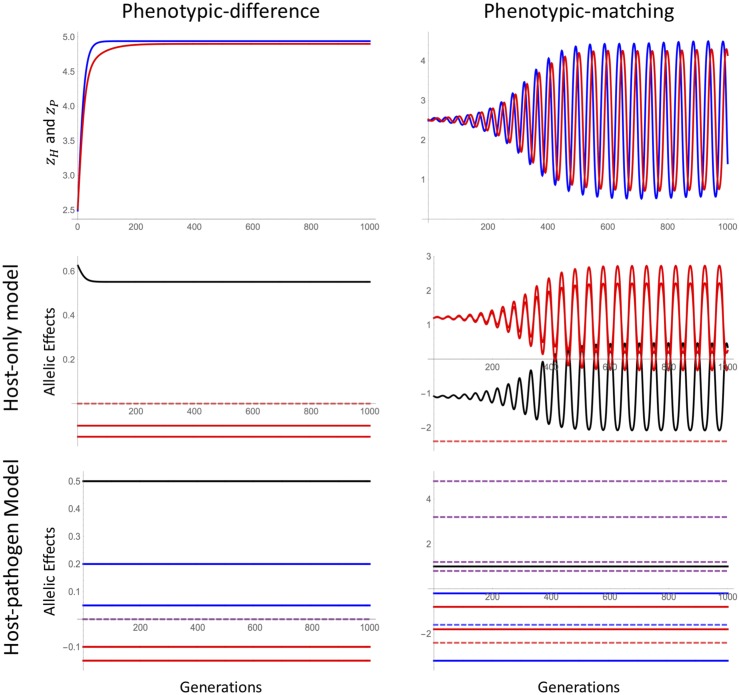
Allelic effects over coevolutionary time. Top row: Phenotypes $z_{H}$ (red) and $z_{P}$ (blue) simulated over coevolutionary time in the phenotypic-difference (left) and phenotypic-matching models (right). Middle row: Coefficients estimated under the host-only model (7) (black is $\beta_{0},$ solid red is $\beta_{Hi},$ dashed red is $\beta_{H1,H2}$). Bottom row: Coefficients estimated under the host–pathogen model (9) (black is $\beta_{0},$ solid red is $\beta_{Hi},$ dashed red is $\beta_{H1,H2},$ blue is $\beta_{Pi},$ dashed blue is $\beta_{P1,P2},$ purple dashed is $\beta_{Hi,Pj}$). Because epistatic and G × G interactions are absent in the phenotypic-difference model, their allelic effects all overlap at 0 and hence are not all visible.

Given these fitnesses, we simulate allele frequencies and linkage disequilibrium over time assuming random mating, a per-locus mutation rate of μ, and a recombination rate *r* (see *Mathematica* notebook in File S1). We then use Equations 7 and 9 to calculate the inferred allelic effect sizes by using a host-only GWAS or co-GWAS for each generation over the course of coevolution for both the phenotypic-difference and phenotypic-matching models (Figure 4).

As expected, using the host-only GWAS approach, the inferred allelic effects can vary over time but only under the quadratic-shaped, phenotypic-matching model. As noted above, the estimated effects can even change sign, having large positive values when sampled in one generation and large negative values when sampled only a few generations later. In contrast, the inferred effects remain constant in the co-GWAS approach regardless of the coevolutionary model. In terms of the phenotypic variation explained, the host-only approach explains only a portion of genetically determined phenotypic variation, whereas the co-GWAS approach can explain up to 100%. The contribution of different genetic components to the total variation explained remains approximately constant under the phenotypic-difference model but varies rapidly as allele frequency changes in the phenotypic-matching model.

### Data availability

The analysis, numerical simulations, and scripts to generate the original figures were coded in Wolfram *Mathematica* 11 (File S1) and are available for download from the Dryad Digital Repository (DOI: https://doi.org/10.5061/dryad.tb25q).

## Daphnia–Pasteuria GWAS

Taken together, our analytical model and simulations illustrate that incorporating pathogen genetic information into the search for disease genes can greatly increase the explanatory power and repeatability of genome scans. Testing these theoretical predictions with biological data is a critical step in evaluating the power of the co-GWAS approach relative to a traditional single-species GWAS. Analysis of biological data will include several complications that we ignored above, including finite sample sizes, arbitrary forms of coevolutionary interactions, and complex genomic architectures. Unfortunately, we know of no studies that include full host and parasite genomic data as well as the outcome of infection experiments. Further, the computational tools to perform a co-GWAS in the form of Equation 8 do not yet exist. We can, however, use recently published data by Bourgeois *et al.* (2017) on the susceptibility of *D. magna* to two *P. ramosa* strains, C1 and C19, as a preliminary test of our analytical predictions. In particular, we compare the results of genome scans for C1 and C19 susceptibility analyzed separately to a single genome scan for susceptibility using all the data but ignoring pathogen strain type. Our analytical model predicts that, despite having half the sample size, the separate genome scans for C1 and C19 resistance should reveal loci that determine host–parasite specificity, whereas the full data scan will have lower power to do so. Note that strain type captures almost all of the relevant genetic information in this case, given that the parasite is clonal.

The original data set, provided on Dryad by the authors (Bourgeois *et al.* 2017), sampled 97 *D. magna* clones from three distinct geographic regions—1 site in Germany, 1 in Switzerland, and 11 sites in Finland—and provided the sequence at 6403 SNPs. Host susceptibility (S: susceptible; R: resistant) infection by each *P. ramosa* strain, C1 and C19, was determined by assessing whether fluorescently labeled spores attached to the host’s esophagus (Duneau *et al.* 2011). All four possible combinations of susceptibility and resistance to the two strains (SS, SR, RS, and RR) were present. By performing two separate association studies, one for each strain, Bourgeois *et al.* (2017) used this experimental design to identify genomic regions associated with susceptibility to a specific parasite strain. Following the methods in the original work, we compare their results to a third genome scan including all the data, a total of 194 samples, ignoring the *Pasteuria* strain type tested. All genome scans were performed using the R package GenAbel, adjusting for population structure and repeated measures of the same host genotype using the Eigenstat method (Aulchenko *et al.* 2007).

**Figure 5:**
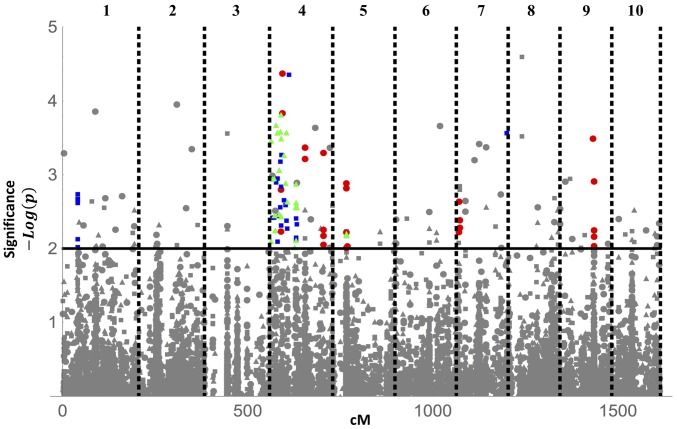
GWAS of *D. magna* susceptibility. Genetic associations of each SNP with C1 (red ●), C19 (blue ▪), and overall susceptibility in the complete data set without parasite-type information (green ▴). Hence, each SNP is represented three times, once for each genome-wide scan. Note that closely linked SNPs often overlap with one another and are not all individually visible. Significant SNPs are shown in color while those below the log-likelihood of two threshold or that are not clustered within a 10-cM region of three other significant SNPs are shown in gray. The 10 linkage groups are delineated by vertical dashed lines.

To accurately assess which genomic regions are associated with susceptibility to C1, C19, and/or “overall” susceptibility from the complete data set, we used the *Daphnia* genetic map constructed by Dukić *et al.* (2016) to array the scaffolds into 10 linkage groups. To limit the detection of false positives, we followed an approach analogous to that used in Bourgeois *et al.* (2017) where SNPs were only considered significantly associated with a given susceptibility phenotype if there existed four SNPs in a 10-cM region with a log-likelihood score >2 (Figure 5). Multiple genomic regions are significantly associated with susceptibility to C1, C19, and to disease susceptibility in the complete data set without strain information. Four linkage groups (4, 5, 7, and 9), with a total of 28 significant SNPs, are associated with C1 susceptibility. Three linkage groups (1, 4, and 7) with 38 SNPs are associated with C19 susceptibility, and two linkage groups (4 and 5) with 35 SNPs are associated with susceptibility in the complete data set. Thus, while the complete data set has twice as many measures of disease susceptibility, it has less power to detect genetic regions underlying disease susceptibility because of the lack of parasite information.

The contrast between the associations for C1 and C19 susceptibility to overall susceptibility in the complete data set provides additional information about the nature of the genetic basis to resistance. Genomic regions associated with the overall resistance regardless of parasite type, particularly when these regions are also associated with C1 and C19 resistance, provide increased resistance regardless of the parasite strain tested and are consistent with general host health and nonspecific immune response. By contrast, sites that are not associated with overall resistance—despite the data set having twice the size—but are associated with either C1 or C19, are good candidates for loci that act in a parasite-specific manner. Examining Figure 5, we therefore conclude that linkage group 4 and possibly 5 are involved in general health and resistance. In contrast, the regions on the far left and right of linkage group 7 as well as the regions on linkage group 1 and 9, which are associated only with C1 or C19 resistance, are indicative of parasite-specific resistance loci.

These conclusions are in agreement with the hypothesized model and previous molecular work on *Daphnia* resistance to *Pasteuria*. In particular, resistance to *Pasteuria* is hypothesized to be controlled by a three-locus, matching-alleles system. One of these loci (the C locus) determines overall host susceptibility regardless of pathogen strain and is thought to reside on linkage group 4 (Bento *et al.* 2017). In the absence of protection from the C locus, a second “A locus” is thought to confer resistance to C1 when the dominant allele is present. The regions detected on linkage groups 7 and 9 in the hosts exposed to C1 may only be candidates for such C1-specific resistance. Finally, if the C locus and A locus are both homozygous recessive, a third “B locus” determines susceptibility to the C19 strain. Such a locus would likely be hard to detect in a GWAS due to epistasis between the A, B, and C loci; nevertheless, the regions associated with only C19 resistance (on linkage groups 1 and 7) would be candidates for such a B locus. Overall we conclude that significant SNPs obtained without accounting for parasite type may signal general health status. Against this background, a co-GWAS can help identify genes whose regions are likely critical to host–parasite specificity and variation in host susceptibility.

## Discussion

Identifying genes that determine a host’s susceptibility to infection is a promising frontier with a wide range of applications, including agriculture and human health. Yet, as our mathematical models demonstrate, association studies focusing on identifying genes in a single species without accounting for the genetics of the interacting species can drastically affect our ability to detect disease genes involved in host–pathogen specificity and limit our ability to account for the genetic variation in disease susceptibility. When the genetic composition of the pathogen population varies over time and/or space, this can further lead to inconsistencies in the results of genetic association studies. Finally, using previously published data on *D. magna* resistance to its *Pasteuria* parasite, we illustrate that performing association studies with and without information about pathogen type can be used to distinguish genomic regions affecting general *vs.* specific resistance to pathogens. Consistent with current models for *Daphania*–*Pasteuria* interactions, we identify one region associated with general health as well as candidate regions more directly involved in mediating host–pathogen specificity.

The mathematical analysis presented above focuses on host–pathogen interactions of a specific form, given by Equation 1. Although we have relied on an approximation that assumes weak phenotype differences, *i.e.*, $z_{H}-z_{P}$ is small, we postulate that the power to detect strain-specific resistance genes will be increased whenever parasite information is incorporated, even when genes have major effects and phenotypic differences become large. Similarly, the methods used above can be extended to include alternative interaction types such as a “matching-alleles” interaction (see *Mathematica* notebook in File S1). The expressions for the *β* coefficients under this interaction model are unruly and difficult to interpret. Using a numerical approach, we observe that once again G × G interactions can explain a significant proportion of the variation in susceptibility (Figure S1 available on Dryad), particularly in highly variable pathogen populations. Unlike the phenotypic-difference and phenotypic-matching models, however, the co-GWAS approach (Equation 8) no longer explains all of the variation in susceptibility and the coefficients vary with pathogen allele frequency. This is a result of higher order interactions not included in our model. Hence, although the co-GWAS approach performs significantly better than a single-species approach, it will not always capture the full genetic basis of infection because of the second order approximation used in Equation 8.

Regardless of the form of the interaction, our analytical models and simulations illustrate that incorporating pathogen genetics into the search for disease genes can greatly increase the explanatory power and repeatability of genome scans. Unfortunately, several logistical and computational challenges preclude applying a full two-species GWAS. Most notably, such a design requires additional genetic data that is not currently available. More specifically, this design requires genotyping all hosts and the pathogens to which they are exposed, not just the host–parasite combinations observed in infected individuals. Future exploration is warranted to determine whether uninfected individuals can simply be treated as unknown with respect to pathogen exposure, and what the consequences of doing so would be for the statistical power of our approach.

The complexity of the two-species design (Equation 8) relative to that of a single-species design (Equation 6) also introduces computational challenges. In addition to requiring larger sample sizes, estimating the effects of the large number of potential G × G interactions in a full host–genome by parasite–genome study is computationally unrealistic. In addition to the large number of pairwise interactions between hosts and pathogens, depending on the form of the interaction, higher order genetic interactions may be necessary to fully explain the variation in susceptibility. These higher order interactions can be particularly important as the number of loci underlying susceptibility, $n_{H}$ and $n_{P},$ increases. Although incorporating complete pathogen genetic data may be unfeasible, there often exists some form of pathogen typing, which is largely indicative of the pathogen’s genotype and may be sufficient for the purposes of a host genome-wide scan. For example, despite its vast diversity, Hepatitis C virus has been subdivided into seven genotypes (Irvine *et al.* 1993; Murphy *et al.* 2015), which may capture much of the relevant variation in host susceptibility.

The *Daphnia*–*Pasteuria* data set we analyzed provides a valuable test case for a two-species co-GWAS. In this study, we know exactly to which pathogen type individuals have been exposed, which is generally not known in natural populations. This information may have increased the power of the study to detect loci underlying C1 and C19 susceptibility. Despite this increased power, we chose to use the arguably lenient significance threshold of a log-likelihood score >2 plus clustering of four or more SNPs, as in the original article. Requiring more stringent threshold corrections for multiple sampling, such as a Bonferroni correction, does not yield any significant SNPs. Given the correspondence between the GWAS results and those of functional studies (Bento *et al.* 2017), however, many of the observed SNPs are arguably not false positives. Using the log-likelihood of two and clustering threshold, we observe fewer genomic regions associated with overall susceptibility when parasite information is not incorporated than when conducting GWAS with exposure to either C1 or C19, despite the complete data set containing twice the number of data points. As an alternative to analyzing the complete data set, we could hold the sample size constant in a combined analysis by randomly choosing whether the host was exposed to C1 or to C19 for each host genotype (Figure S2 available on Dryad). Interestingly, this “mixed” GWAS not only identifies the same regions on linkage group 4 and 5 but also identifies regions on linkage groups 1, 9, and 10, as were found in the single pathogen-type GWAS. The fact that this mixed analysis picks up some of the potentially parasite-specific loci is likely due to randomly sampling an excess of C1- or C19-tested clones. Consistent with this interpretation, exactly which parasite-specific regions are identified varies with the random sample chosen. Nevertheless, as with the complete data set, a comparison between C1, C19, and mixed susceptibility provides additional information about which genes are involved in general health *vs.* parasite-specific susceptibility.

The results presented here highlight several important avenues for future research. First and foremost, designing genome-wide association methods that allow for G × G interactions is critically important, as is the collection of genotypic data from hosts and pathogens. This could be approached, for example, by adapting GWAS designs and analyses used to detect gene-by-environment interactions (Winham and Biernacka 2013). Recognizing the importance of host–pathogen genetic interactions is important for understanding the applicability and limitations of single-species association scans. Developing metrics that capture relevant variability in host and pathogen populations may facilitate the application of these results. Finally, incorporating G × G interactions into our association studies will also enable us to understand what mathematical models of host–parasite interactions best predict the genetic interactions observed in natural systems, allowing for further refinements of the models.

## Supplementary Material

Supplemental material is available online at www.genetics.org/lookup/suppl/doi:10.1534/genetics.117.300481/-/DC1.

## References

[bib1] Anagnostakis S., 2000 Revitalization of the majestic chestnut: chestnut blight disease. APSnet Features. Online. DOI: 10.1094/APSnetFeature-2000-1200 .

[bib2] Anderson P. K. Cunningham A. A. Patel N. G. Morales F. J. Epstein P. R., 2004 Emerging infectious diseases of plants: pathogen pollution, climate change and agrotechnology drivers. Trends Ecol. Evol. 19: 535–544. 16701319 10.1016/j.tree.2004.07.021

[bib3] Ashby B. Boots M., 2017 Multi-mode fluctuating selection in host-parasite coevolution. Ecol. Lett. 20: 357–365. 28133876 10.1111/ele.12734

[bib4] Aulchenko Y. S. Ripke S. Isaacs A. van Duijn C. M., 2007 GenABEL: an R library for genome-wide association analysis. Bioinformatics 23: 1294–1296. 17384015 10.1093/bioinformatics/btm108

[bib5] Bartholomew J. L., 1998 Host resistance to infection by the myxosporean parasite *Ceratomyxa shasta*: a review. J. Aquat. Anim. Health 10: 112–120.

[bib6] Bayne C. J., 2009 Successful parasitism of vector snail *Biomphalaria glabrata* by the human blood fluke (trematode) *Schistosoma mansoni*: a 2009 assessment. Mol. Biochem. Parasitol. 165: 8–18. 19393158 10.1016/j.molbiopara.2009.01.005 PMC2765215

[bib7] Bento G. Routtu J. Fields P. D. Bourgeois Y. Du Pasquier L., 2017 The genetic basis of resistance and matching-allele interactions of a host-parasite system: the *Daphnia magna-Pasteuria ramosa* model. PLoS Genet. 13: e1006596. 28222092 10.1371/journal.pgen.1006596 PMC5340410

[bib8] Bourgeois Y. Roulin A. C. Müller K. Ebert D., 2017 Parasitism drives host genome evolution: insights from the *Pasteuria ramosa-Daphnia magna* system. Evolution. 71: 1106–1113. 28230237 10.1111/evo.13209

[bib9] Chapman S. J. Hill A. V. S., 2012 Human genetic susceptibility to infectious disease. Nat. Rev. Genet. 13: 175–188. 22310894 10.1038/nrg3114

[bib10] Dukić M. Berner D. Roesti M. Haag C. R. Ebert D., 2016 A high-density genetic map reveals variation in recombination rate across the genome of *Daphnia magna*. BMC Genet. 17: 137. 27737627 10.1186/s12863-016-0445-7 PMC5064971

[bib11] Duneau D. Luijckx P. Ben-Ami F. Laforsch C. Ebert D., 2011 Resolving the infection process reveals striking differences in the contribution of environment, genetics and phylogeny to host-parasite interactions. BMC Biol. 9: 11. 21342515 10.1186/1741-7007-9-11 PMC3052238

[bib12] Gurung S. Mamidi S. Bonman J. M. Xiong M. Brown-Guedira G., 2014 Genome-wide association study reveals novel quantitative trait loci associated with resistance to multiple leaf spot diseases of spring wheat. PLoS One 9: e108179. 25268502 10.1371/journal.pone.0108179 PMC4182470

[bib13] Irvine B. Yap P. L. Kolberg J. Chan S.-W. Cha T.-A., 1993 Classification of hepatitis C virus into six major genotypes and a series of subtypes by phylogenetic analysis of the NS-5 region. J. Gen. Virol. 74: 2391–2399. 8245854 10.1099/0022-1317-74-11-2391

[bib14] Johnson N. P. A. S. Mueller J., 2002 Updating the accounts: global mortality of the 1918–1920 “Spanish” influenza pandemic. Bull. Hist. Med. 76: 105–115. 11875246 10.1353/bhm.2002.0022

[bib15] Khor C. C. Hibberd M. L., 2012 Host-pathogen interactions revealed by human genome-wide surveys. Trends Genet. 28: 233–243. 22445588 10.1016/j.tig.2012.02.001

[bib16] Kopp M. Gavrilets S., 2006 Multilocus genetics and the coevolution of quantitative traits. Evolution 60: 1321–1336. 16929650

[bib17] Lambrechts L., 2010 Dissecting the genetic architecture of host-pathogen specificity. PLoS Pathog. 6: e1001019. 20700450 10.1371/journal.ppat.1001019 PMC2916863

[bib18] Manolio T. A. Collins F. S. Cox N. J. Goldstein D. B. Hindorff L. A., 2009 Finding the missing heritability of complex diseases. Nature 461: 747–753. 19812666 10.1038/nature08494 PMC2831613

[bib19] Mitta G. Adema C. M. Gourbal B. Loker E. S. Theron A., 2012 Compatibility polymorphism in snail/schistosome interactions: from field to theory to molecular mechanisms. Dev. Comp. Immunol. 37: 1–8. 21945832 10.1016/j.dci.2011.09.002 PMC3645982

[bib20] Mon Y. Ribou A.-C. Cosseau C. Duval D. Thron A., 2011 An example of molecular co-evolution: reactive oxygen species (ROS) and ROS scavenger levels in *Schistosoma mansoni/Biomphalaria glabrata* interactions. Int. J. Parasitol. 41: 721–730. 21329695 10.1016/j.ijpara.2011.01.007

[bib21] Murphy D. G. Sablon E. Chamberland J. Fournier E. Dandavino R., 2015 Hepatitis C virus genotype 7, a new genotype originating from central Africa. J. Clin. Microbiol. 53: 967–972. 25520447 10.1128/JCM.02831-14 PMC4390628

[bib22] Newport M. J. Finan C., 2011 Genome-wide association studies and susceptibility to infectious diseases. Brief. Funct. Genomics 10: 98–107. 21436306 10.1093/bfgp/elq037

[bib23] Nuismer S. L. Ridenhour B. J. Oswald B. P., 2007 Antagonistic coevolution mediated by phenotypic differences between quantitative traits. Evolution 61: 1823–1834. 17683426 10.1111/j.1558-5646.2007.00158.x

[bib24] Ratcliffe F. N. Myers K. Fennessy B. V. Calaby J. H., 1952 Myxomatosis in Australia: a step towards the biological control of the rabbit. Nature 170: 7–11. 14957004 10.1038/170007a0

[bib25] Revers F. Nicaise V., 2014 Plant resistance to infection by viruses, in Encyclopedia of Life Sciences. John Wiley & Sons, Ltd, Chichester, UK.

[bib26] Rowell J. L. Dowling N. F. Yu W. Yesupriya A. Zhang L., 2012 Trends in population-based studies of human genetics in infectious diseases. PLoS One 7: e25431. 22347358 10.1371/journal.pone.0025431 PMC3274513

[bib27] Taubenberger J. K. Morens D. M., 2006 1918 influenza: the mother of all pandemics. Emerg. Infect. Dis. 12: 15–22. 16494711 10.3201/eid1201.050979 PMC3291398

[bib28] Thomas D., 2010 Gene-environment-wide association studies: emerging approaches. Nat. Rev. Genet. 11: 259–272. 20212493 10.1038/nrg2764 PMC2891422

[bib29] Wang M. Yan J. Zhao J. Song W. Zhang X., 2012 Genome-wide association study (GWAS) of resistance to head smut in maize. Plant Sci. 196: 125–131. 23017907 10.1016/j.plantsci.2012.08.004

[bib30] Weatherall D. J. Clegg J. B., 2002 Genetic variability in response to infection: malaria and after. Genes Immun. 3: 331–337. 12209359 10.1038/sj.gene.6363878

[bib31] Winham S. J. Biernacka J. M., 2013 Gene-environment interactions in genome-wide association studies: current approaches and new directions. J. Child Psychol. Psychiatry 54: 1120–1134. 23808649 10.1111/jcpp.12114 PMC3829379

[bib32] Zila C. T. Samayoa L. F. Santiago R. Butrón A. Holland J. B., 2013 A genome-wide association study reveals genes associated with fusarium ear rot resistance in a maize core diversity panel. Genetics 3: 2095–2104. 10.1534/g3.113.007328 PMC3815068 24048647

