# Supplementary figures and images for: Keeping Pace with the Red Queen: Identifying the Genetic Basis of Susceptibility to Infectious Disease
Source: Genetics. 2017 Dec 8;208(2):779–89. doi: 10.1534/genetics.117.300481 (PMC5788537; doi:10.1534/genetics.117.300481)

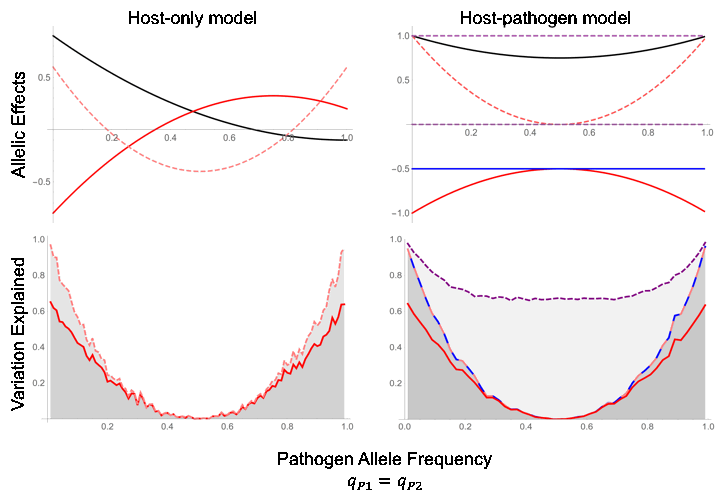

Supplement: Supplementary file 1 [file 779FigureS1.gif]

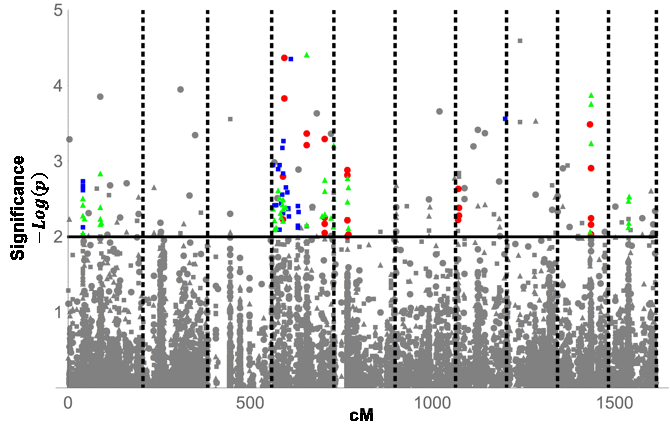

Supplement: Supplementary file 2 [file 779FigureS2.gif]
